# Supplementary figures and images for: A type 2 diabetes disease module with a high collective influence for Cdk2 and PTPLAD1 is localized in endosomes
Source: PLoS One. 2018 Oct 9;13(10):e0205180. doi: 10.1371/journal.pone.0205180 (PMC6177195; doi:10.1371/journal.pone.0205180)

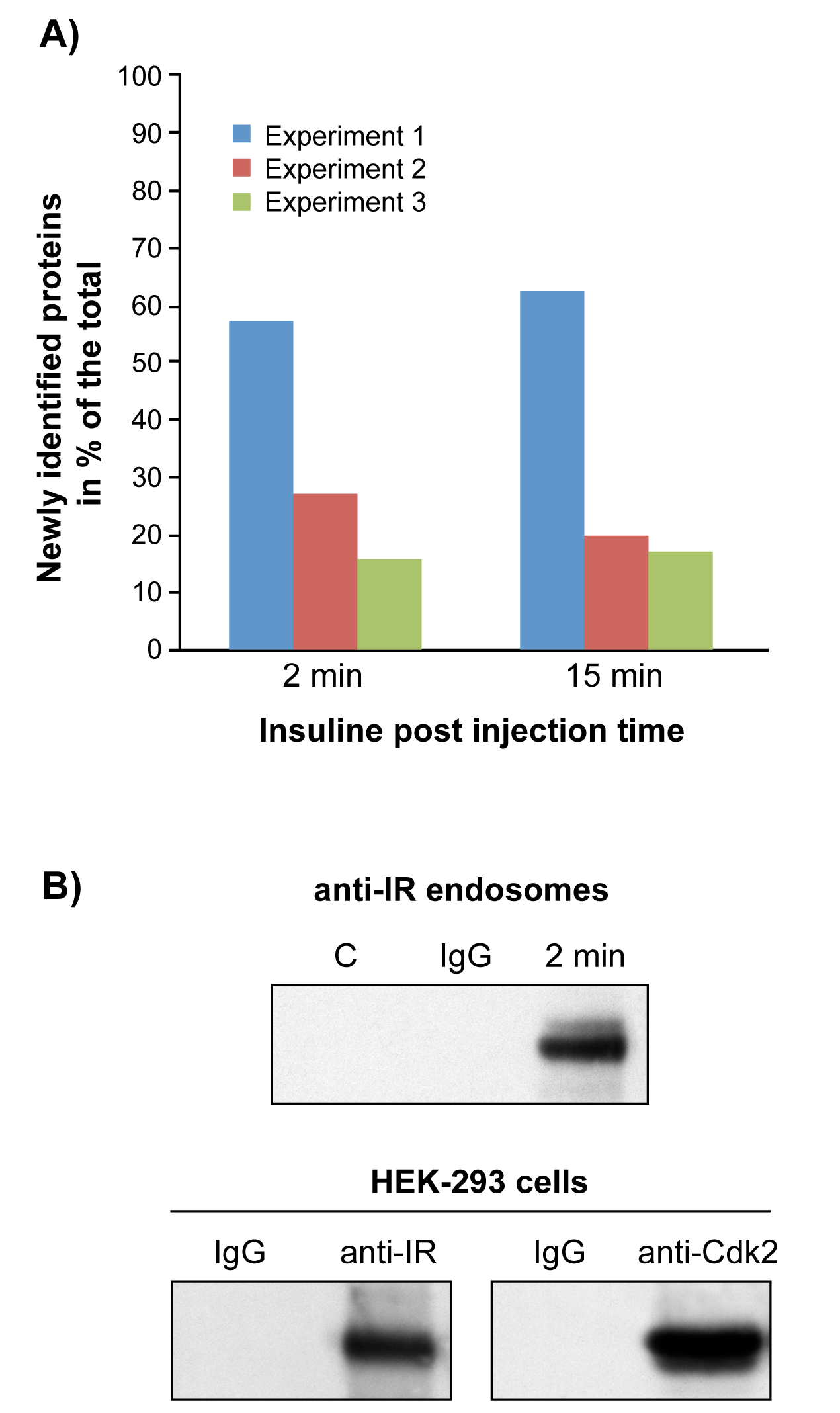

Supplement: S1 Fig — A) IREP, the number of newly identified proteins from one independent experiment to another (tryptic peptides, see Methods). B) upper panel: The G/E fraction was prepared 2 minutes after insulin injections and incubated with uncoated beads (C), with beads coated with an unrelated IgG (IgG) or beads coated with the anti-IR (2 min). The bound fraction was blotted and incubated with the antibody against IR β-subunit. Lower panel: HEK293 cells were preincubated in serum-free medium for 5 hours and then stimulated for the indicated times with insulin (35 nM). Left, IP with an unrelated IgG (IgG) or the anti-IR antibody (anti-IR). Right, IP with an unrelated IgG (IgG) or the anti-Cdk2 antibody. (TIF) [file pone.0205180.s001.tif]

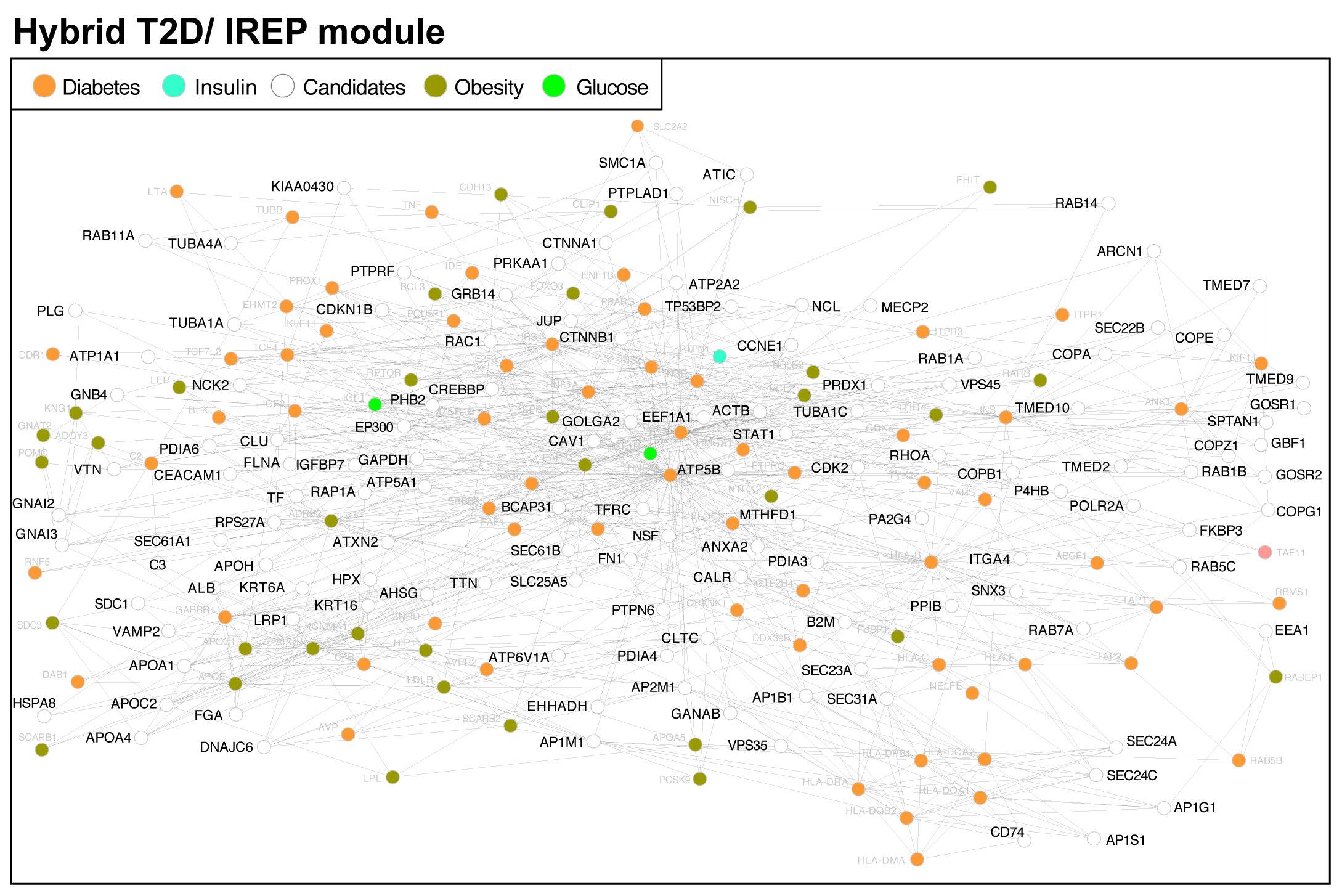

Supplement: S2 Fig — In total, 126 IREP proteins were selected on the basis of having each at least three interactors among 112 of the 184 seeds of the T2D protomodule (S4 Table-sheet hybrid module). The 126 IREP coding genes make up the list of candidates based on the PPIN approach. The diabetes-associated genes (DAGs) are represented according to the colors indicated in the legend. Orange: Diabetes-associated traits. Blue: Insulin-associated traits. Dark green: Obesity-associated traits. Green: Glycemic traits (S3 Table-sheet validated DAGs seeds). White: IREP candidates. (TIF) [file pone.0205180.s002.tif]

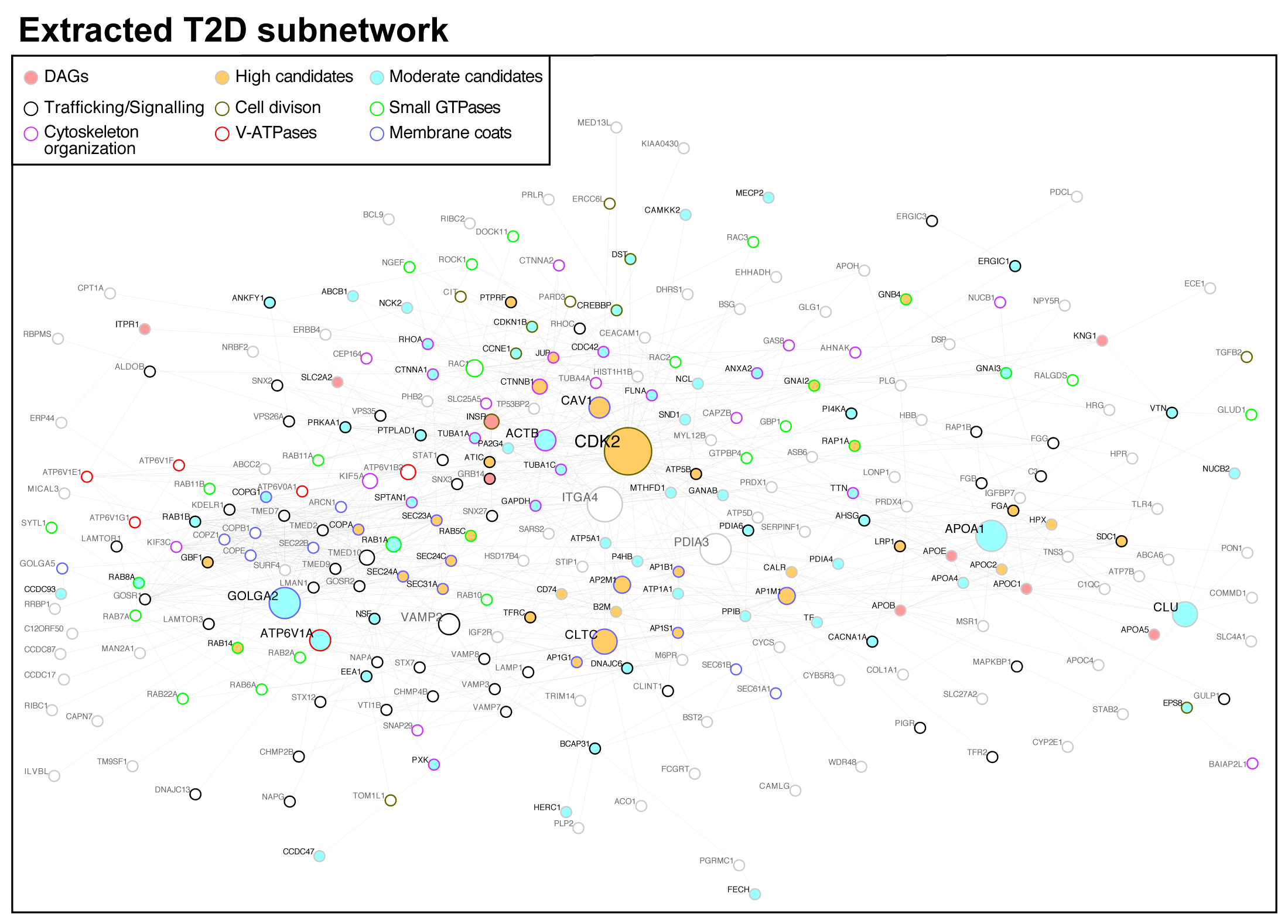

Supplement: S3 Fig — The general topology of IREN is conserved and based on few major hubs, with the kinase Cdk2 displaying the highest centrality (S8 Table). Candidates (yellow and blue colors and black characters; Tables 1 and 2) and DAGs (pink color and black characters) form a single-connected disease module of 94 nodes (33% of IREN nodes) with 330 interactions (28,7% of IREN interactions). An expansion to the first level of adjacent nodes results in a connected subnetwork of 272 nodes (88% of nodes) covering 92% of interactions (1070 out of 1147 IREN interactions). The functional groups are represented according to the colors of borders indicated in the legends. (TIF) [file pone.0205180.s003.tif]

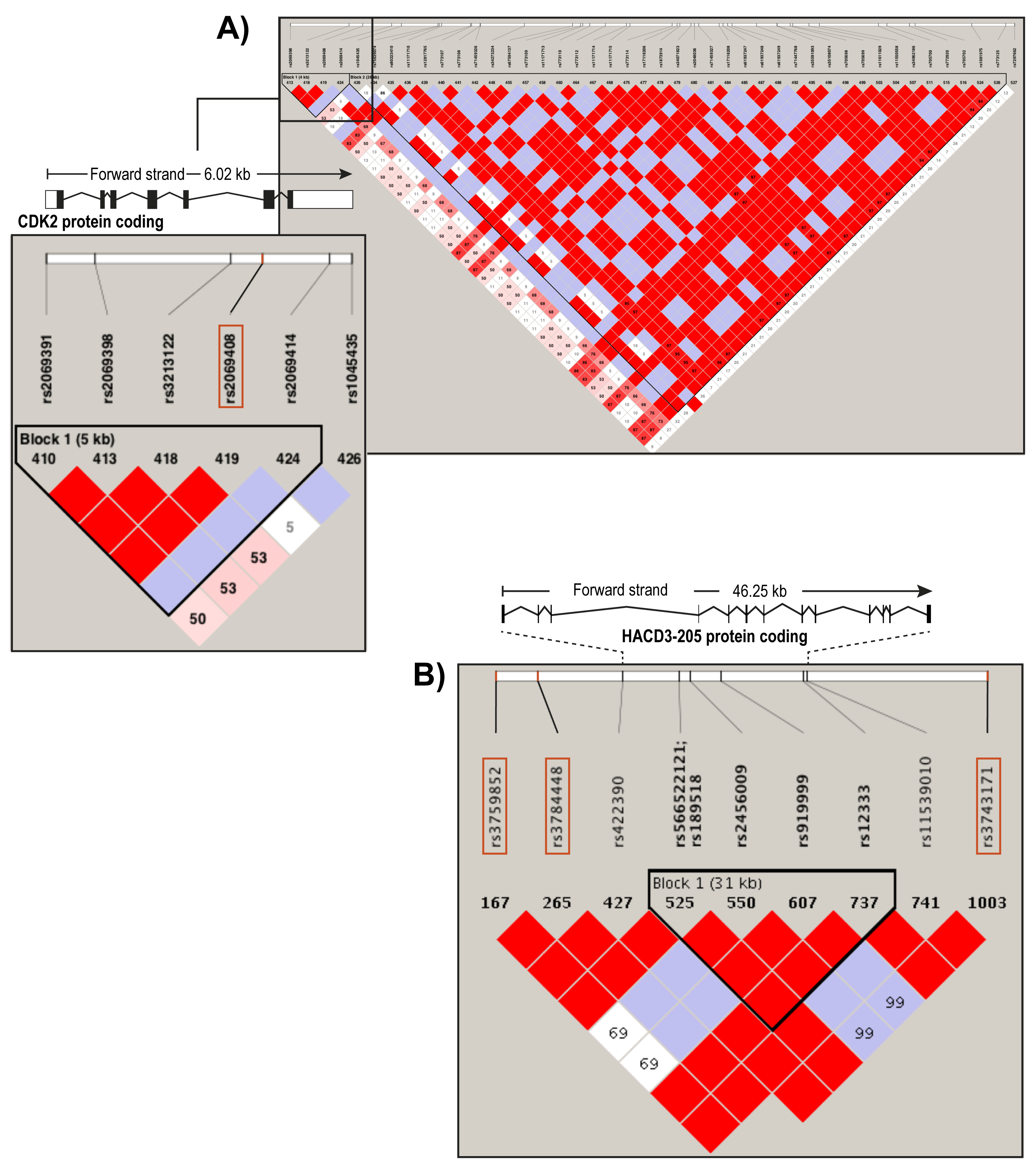

Supplement: S4 Fig — For all graphs, a reference track with chromosomal location, SNP position, and gene position runs along the top. (Red) indicates strong LD between markers; (white) no LD; (light blue) lack of information to evaluate LD. The block-like patterns of LD are evident in the triangles representing regions of high LD, divided by narrow areas where even adjacent markers are completely independent. The SNP in the red box are associated with T2D. A) Cdk2 genotyped in European population (CEU TSI FIN GBR) as part of the 1000 Genomes project (release 2013/05/03). The pop-up represents the haplotype block of the SNP rs2069408 implicated in T2D (S2 Table). B) The HACD3 genotype in an African population (YRI) as part of the 1000 Genomes project (release 2013/05/03). Three SNPs associate with T2D (rs3759852, rs3784448 and rs3743171, S2 Table) and SNPs in strong LD with these three SNPs in the gene HACD3 (PTPLAD1). (TIF) [file pone.0205180.s004.tif]

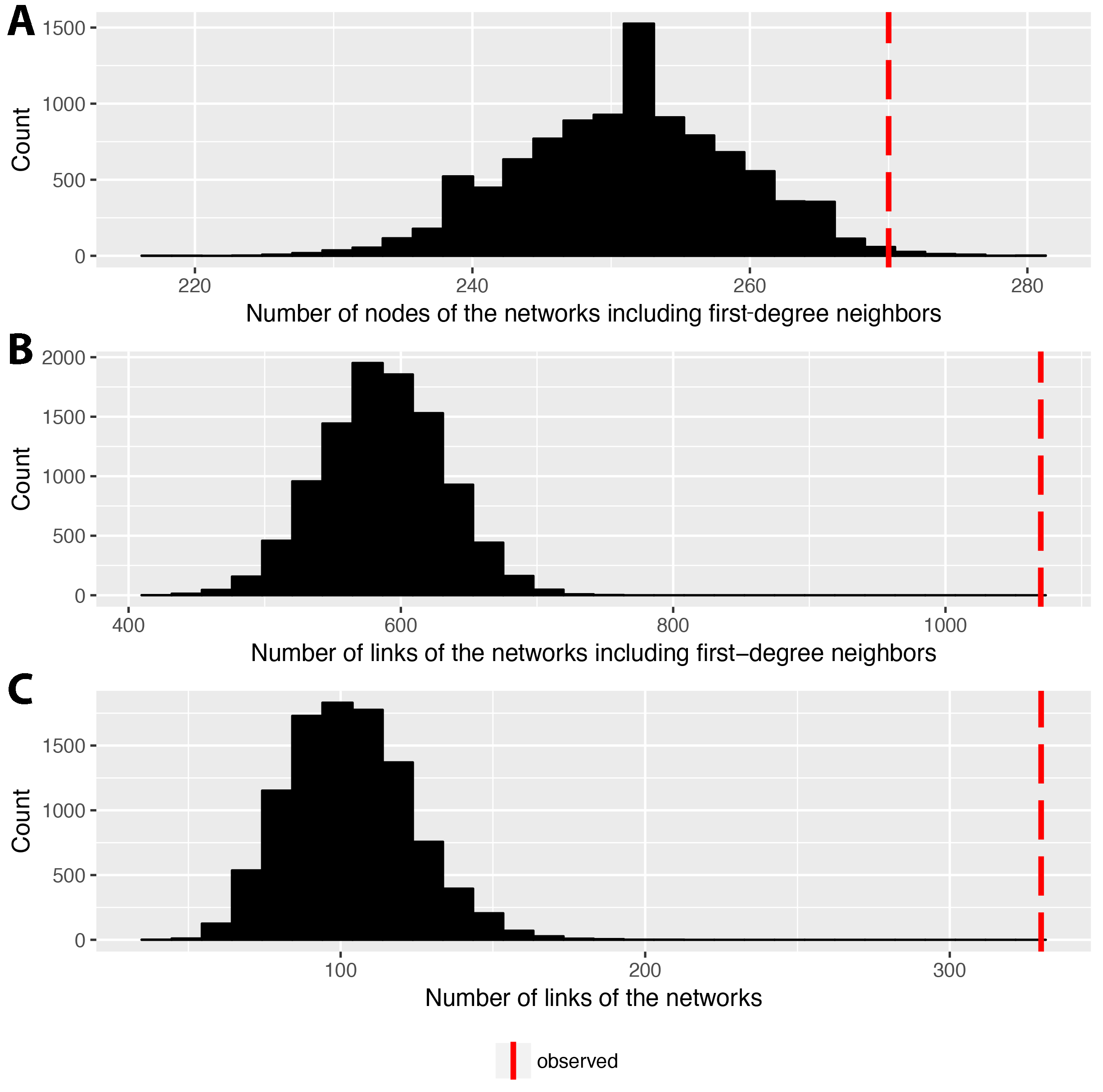

Supplement: S5 Fig — Subnetworks were constructed by 10 000 reiterations of 94 randomly selected IREN nodes. For each simulation, the number of interactions was computed protocols.iodx.doi.org/10.17504/protocols.io.sdqea5w). A) The distribution of the number of nodes for each subnetwork with the neighborhood of 94 selected nodes; the number of nodes observed with the T2D subnetwork is in red. B) The distribution of the number of interactions in the same subnetwork used in A; in red, the number of interaction observed with the T2D subnetwork. C) Distribution of the number of interactions; the number of interactions observed from the 94 T2D nodes is in red. (TIF) [file pone.0205180.s005.tif]
